# Oleanolic Acid-Enriched Olive Oil Alleviates the Interleukin-6 Overproduction Induced by Postprandial Triglyceride-Rich Lipoproteins in THP-1 Macrophages

**DOI:** 10.3390/nu13103471

**Published:** 2021-09-29

**Authors:** Ángel Fernández-Aparicio, Javier S. Perona, José M. Castellano, María Correa-Rodríguez, Jacqueline Schmidt-RioValle, Emilio González-Jiménez

**Affiliations:** 1 Department of Nursing, Faculty of Health Sciences, University of Granada, Av. Ilustración, 60, 18016 Granada, Spain; anfeapa@ugr.es (Á.F.-A.); macoro@ugr.es (M.C.-R.); jschmidt@ugr.es (J.S.-R.); emigoji@ugr.es (E.G.-J.); 2 Department of Food and Health, Instituto de la Grasa-CSIC, Campus of the University Pablo de Olavide, Building 46, 41013 Seville, Spain; jmcas@ig.csic.es

**Keywords:** oleanolic acid, olive oil, functional foods, postprandial trial, human triglyceride-rich lipoproteins, metabolic syndrome, obesity, insulin resistance, adolescents, THP-1 macrophages

## Abstract

Oleanolic acid (OA), a triterpene that is highly present in olive leaves, has been proposed as a component of functional foods for the prevention of metabolic syndrome, due to its anti-inflammatory activity. We analyzed the effects of OA on inflammatory parameters and signaling proteins in LPS-stimulated THP-1 macrophages. Thus, THP-1 macrophages were incubated with LPS for 48 h after pretreatment with OA at different concentrations. Pretreatment with OA was significantly effective in attenuating IL-6 and TNF-α overproduction induced by LPS in macrophages, and also improved the levels of AMPK-α. We also evaluated the effects of human triglyceride-rich lipoproteins (TRLs) derived from individuals consuming an OA-enriched functional olive oil. For this purpose, TRLs were isolated from healthy adolescents before, 2 and 5 h postprandially after the intake of a meal containing the functional olive oil or common olive oil, and were incubated with THP-1 macrophages. THP-1 macrophages incubated with TRLs isolated at 2 h after the consumption of the OA-enriched olive oil showed significant lower levels of IL-6 compared to the TRLs derived from olive oil. Our results suggest that OA might have potential to be used as a lipid-based formulation in functional olive oils to prevent inflammatory processes underlying metabolic syndrome in adolescents.

## 1. Introduction

Metabolic syndrome (MetS) is a worrying health public problem that affects approximately 31% of the world population, and it is expected that its prevalence will increase by about 53% by 2035 [1,2]. The International Diabetes Federation defines central obesity as the unique fixed component of MetS [3]. In fact, inadequate control of abdominal obesity and insulin resistance (IR), among others, not only lead to the development of Type 2 Diabetes Mellitus (T2DM) and cardiovascular diseases, but also to other clinical disorders, such as oxidative stress, non-alcoholic fatty liver disease and hepatic steatosis [4,5,6].

Obesity is characterized by an excess of fat accumulation in the adipose tissue [7], in which the inflammatory response is closely linked to the development of IR [8]. Chronic inflammation of adipose tissue in obesity causes an impairment in the polarization of adipose tissue macrophages (ATMs), resulting in a higher presence of the M1 proinflammatory phenotype [8,9,10]. As a consequence, ATMs are involved in the release of proinflammatory cytokines, such as interleukin-6 (IL-6), interleukin-1β (IL-1β) and tumor necrosis factor-α (TNF-α) [11,12]. These proinflammatory cytokines are positively regulated by the nuclear transcription factor kappa B (NF-κB), which is translocated to the cell nucleus after activation of IκB kinase (IKK). Another kinase with an important role in NF-κB function is AMP-activated protein kinase (AMPK), in which its activation inhibits NF-κB signaling. Both AMPK and IKK also participate in insulin signaling through acting on the insulin receptor substrate [13]. These pathways that are associated with obesity-induced inflammation have been considered as potential targets in the research of new bioactive compounds or drugs for treating obesity and IR, two of the main factors underlying MetS.

Sedentary and unhealthy lifestyles, both in adults and in adolescents, makes the search of new preventive and therapeutic interventions to fight MetS necessary [14]. Moreover, the worrying comorbidities derived from MetS together with the expensive tendency of the pharmacological interventions used for preventing and controlling this syndrome jeopardize the stability of the healthcare systems [15]. The Seven Country Study accomplished in the 1950s first proved the cardioprotective abilities and health benefits of the Mediterranean diet [16]. Since then, intervention studies based on the Mediterranean diet have gained importance for their beneficial effects on the components of MetS, such as obesity, hyperlipidemia, hyperglycemia and hypertension [17]. The key constituent of the Mediterranean diet is virgin olive oil, which possesses anti-inflammatory and antioxidant properties that are not only due to the high levels of oleic acids, but also to the presence of minor compounds, such as phenolic derivatives, phytosterols, carotenoids, tocopherols and terpenoids [18,19].

There is increasing interest in performing clinical trials to study the preventive and therapeutic effects of virgin olive oil minor compounds on MetS. Sánchez-Rodríguez et al. [20] have recently presented the results of a randomized controlled trial with 51 healthy individuals who consumed a functional olive oil enriched in their phenolics and triterpenes for three weeks, showing that this dietary intervention decreased urinary DNA oxidation and plasma inflammatory biomarkers, in comparison with the intake of a standard virgin olive oil. In addition, the PREDIABOLE study, a randomized controlled trial with 176 prediabetic patients demonstrated that the consumption of an oleanolic acid-enriched olive oil for 30 months improved insulin resistance and diminished the risk of developing T2DM by 55% [21]. Oleanolic acid (OA; (3β)-3-hydroxyolean-12-en-28-oic acid) is a pentacyclic triterpene widely distributed in the plant kingdom, which is significantly abundant in the fruit and leaf from the olive tree [22], and is therefore a natural component of virgin olive oil [23]. This triterpene possesses anti-inflammatory, anti-oxidant, anti-atherosclerotic and anti-hypertensive activities [22,24], but the pathways involved in these properties are not fully understood. However, it is suggested that OA could be entangled in the repression of the NF-κB and in the activation of the nuclear factor erythroid 2–related factor 2 (Nrf2), two important transcription factors that modulate the inflammatory status in insulin resistance [25,26].

Therefore, a better understanding of the action of OA in a metabolic syndrome context is still necessary. For this reason, our study aimed to evaluate the effects of OA on the production of the pro-inflammatory cytokines and on signaling proteins in LPS-stimulated THP-1 macrophages. We also studied for the first time the potential capacity of OA to be used in functional foods in order to prevent IR and MetS, by assessing the effects of postprandial TRLs obtained from healthy adolescents after the intake of a meal containing an OA-enriched olive oil.

## 2. Materials and Methods

### 2.1. Materials

A scanning multi-well spectrophotometer (Multiskan spectrum, Thermo Fisher Scientific, Waltham, MA, USA) was utilized to measure absorbances. RPMI 1640 medium, fetal bovine serum (FBS), penicillin, streptomycin and phosphate buffered saline (PBS) were purchased from Biowest (Nuaillé, France). Lipopolysaccharide (LPS) (from E. coli 0111:B4) and phorbol 12-myristate-13-acetate (PMA) were purchased from Sigma-Aldrich (St. Louis, MO, USA).

### 2.2. OA Obtaining and Elaboration of the Functional Olive Oil

#### 2.2.1. OA Obtaining

OA was obtained from olive tree leaves following the method developed by Albi et al. [27]. In brief, OA was extracted from leaves using 96% ethanol (20 mL/g) at room temperature. To facilitate the formation of OA crystals, the extract was filtered and vacuum concentrated. OA crystals were washed with cold 96% ethanol (5–7 °C) and filtered to remove pigment traces and other possible contaminants. Finally, the crystals were heated at 165 °C, and homogenized to a powder. OA purity was determined by gas chromatography.

#### 2.2.2. Analysis of OA by Gas Chromatography (GC)

Prior to derivatization, 100 μL of a methanolic solution of betulinic acid (0.5 mg/mL) was added as an internal standard to 100 μL of the OA-containing sample. The mixture was evaporated to dryness under a N_2_ stream, and the residue dissolved in 200 μL of the silylating reagent (BSTFA + 1% TMCS in pyridine).

OA was identified and quantified using a coupled GC–mass spectrometry detector (GC-MS) QP2010 Ultra (Shimadzu Europa GmbH, Duisburg, Germany) with an AOC-20i autosampler, an ion source of electron impact and a quadrupole detector. The analysis was carried out in splitless mode, with an injector temperature of 290 °C. Helium was used as a carrier gas at 53.1 kPa and of 1mL/min. The oven temperature program was as follows: initial temperature, 50 °C/1min; 50–200 °C at 40 °C/min; 200–280 °C at 10 °C/min; and finally held for 2 min. Total run time was 14.75 min. The MS conditions were: interface temperature, 280 °C; ion source temperature, 220 °C; electron impact, 70 eV; acquisition mode, scan (*m*/*z* 50–600). OA was identified by comparing the retention times and abundance ratios of two fragments ions (203 and 189 *m*/*z)*.

### 2.3. Elaboration and Characteristics of the Functional Olive Oil

The functional olive oil used in the postprandial trial was elaborated by adding OA at a concentration of 610 mg/kg to a commercial olive oil. No adjuvants were added. The control olive oil was the same as that of the functional olive oil, but without adding OA. Green-colored flasks were used for bottling of the functional and non-functional olive oils, and were labeled with a bicolor code system for blinding. Each dose of the functional oil consisted of 55 mL, containing 30 mg of OA.

Representative chemical compositions of the oils are shown in Table S1 (see the supplementary materials). OA concentrations in the control and in the functional olive oil were 3.8 ± 0.1 mg/kg oil and 610.4 ± 16.2 mg/kg oil, respectively.

### 2.4. Preparation of OA Used in the LPS-Stimulated THP-1 Macrophages

When OA was directly administered to LPS-stimulated THP-1 macrophages, it was dissolved in DMSO. To achieve the concentrations of 0.5, 1, 5, 10 and 25 µM, increased volumes of this solution were employed. Thus, for higher OA concentrations, higher volumes of DMSO were added to cells.

### 2.5. Postprandial Trial

#### 2.5.1. Design and Implementation of the Study

We performed a randomized, controlled and double-blind postprandial trial with adolescents to obtain postprandial TRLs as circulating OA vehicle when the triterpene was administered as functional olive oil. The study was carried out in accordance with the Helsinki declaration and authorized by the research ethics committee of the University Hospitals Virgen de la Macarena and Virgen del Rocío in Seville, Spain. This trial was registered at ClinicalTrials.gov (Identifier: NCT05049304).

#### 2.5.2. Setting and Participants

Twenty-two healthy and normal weight adolescents of both sexes (aged 16–17 years-old) participated in the postprandial trial. The participants were recruited from a high school in the province of Granada (Spain), and their health status was checked through a complete biochemical analysis. Participants did not suffer from digestive, metabolic or oncologic disorders, or any other pathology. For their inclusion in the trial, it was also required that their parents granted the written consent to the approved protocols, after being conveniently informed both orally and by written means. The fieldwork was performed in the high school. Eleven adolescents were randomly assigned to the intervention group (OA-enriched functional olive oil), and the other eleven participants to the control one (the same commercial olive oil not enriched in OA).

The determination of the sample size for the postprandial trial was calculated considering plasma OA concentration as the quantitative variable. As there are no population data, a cohort of 60 participants from the control group of the PREDIABOLE study was used. A confidence of 95% (α risk = 0.05; 1.645) and a power of 90% (zβ = 1.282), were used, yielding a sample size of 15 participants. Taking into account possible losses, sample size was finally augmented to 22 participants.

#### 2.5.3. Anthropometric Assessment and Body Composition Analysis

The intervention was carried out after 12 h of overnight fasting. Anthropometric measurements were performed individually according to the recommendations of the International Society for the Advancement of Kinanthropometry (ISAK) [28] in a classroom provided by the high school to guarantee the privacy of each adolescent. We used a body composition analyzer (TANITA^®^ model BC-418MA, West Drayton, UK) to measure the body weight and the percentage of body fat. Height was measured twice with a ±5 mm precision with a Seca 214* portable stadiometer, and the average of the two values was used in the analysis. The body mass index (BMI) was obtained by dividing the body weight in kilograms by the height in meters squared. We measured waist circumference (WC) in the middle distance from the lowest rib to the upper border of the iliac crest at the end of a normal exhalation using a Seca automatic roll-up measuring tape (precision of 1 mm). Blood pressure (BP) levels were determined using a calibrated aneroid sphygmomanometer and a Littmann^®^ stethoscope (Saint Paul, MN, USA), according to international recommendations [29]. Adolescents self-reported their pubertal development based on the Tanner stages [30].

#### 2.5.4. Intervention

On the trial day, and after 12 h of overnight fasting, blood samples were drawn from the cubital vein. Subsequently, participants took an experimental breakfast that consisted of 150 mL skimmed milk, a tablespoon of crushed tomato, three slices of whole meal bread, and 55 mL of the assigned oil (equivalent dose of OA 30 mg, in the case of the functional olive oil). After eating the respective breakfasts, aliquots of cubital blood were drawn at 2 and 5 h in the postprandial period. During that time, free access to water intake was allowed.

#### 2.5.5. Biochemical Determinations and Isolation of TRLs

Aliquots of cubital blood samples were drawn before, and at 2 and 5 h after the breakfast intake. All blood samples were centrifuged at 3000 rpm for 5 min at 20 °C, and aliquots of serum were frozen until their use for determining biochemical parameters at baseline or isolating TRLs.

Serum levels of fasting glucose, high-density lipoprotein cholesterol (HDL-c), total cholesterol (TC) and triglycerides (TG) were measured by means of enzymatic colorimetric methods. The Friedewald equation ((LDLC) = (Total Cholesterol) − (HDL-c) − ([TG]/5)) was used for estimating low-density lipoprotein cholesterol (LDL-c) [31]. Fasting insulin serum levels were determined using an ELISA kit (Diaclone, Besançon, France) according to the manufacturer’s instructions. HOMA-IR was calculated using the following equation: fasting glycemia (mmol/L) × fasting insulin (mU/L)/22.5.

Once thawed, 4–4.5 mL of serum was layered under 6 mL of 0.9% NaCl solution (d = 1.006 Kg/L), and ultracentrifuged at 39.000 rpm for 16 h at 12 °C in a preparative ultracentrifuge Beckman Coulter L90K (Beckman Instruments, Inc., Palo Alto, CA, USA). Finally, the upper off-white phase of each tube, containing TRLs, was collected.

### 2.6. THP-1 Monocytes Culture

THP-1 monocytes (3–8 × 10^5^ cells/mL) were cultured in RPMI-1640 medium supplemented with antibiotics (100 U/mL streptomycin and 100 U/mL penicillin) and 10% heat-inactivated fetal bovine serum (FBS), under a 100% humidified atmosphere of air + 5% CO_2_ at 37 °C. Cells were passaged every 2 to 3 days to maintain growth.

### 2.7. Differentiation of THP-1 Monocytes into Macrophages

THP-1 monocytes were seeded at 1 × 10^6^ cells/mL in cell culture dishes, and incubated with PMA (0.2 µg/mL) at 37 °C in 5% CO_2_–95% air to induce their differentiation into macrophages. After incubation for 72 h, cells were washed with preheated RPMI-1640 medium supplemented with antibiotics to eliminate the excess of PMA and undifferentiated monocytes. The confluency of adherent cells was 70–80% in all cell-culture experiments, and cell viability was >95%. Finally, THP-1 macrophages were maintained with RPMI-1640 medium supplemented with antibiotics for 24 h at 37 °C in 5% CO_2_–95% air, until the addition of the experimental (OA or TRL) or vehicle.

### 2.8. Cell Viability Assay in THP-1 Macrophages Pretreated with OA

THP-1 macrophages were pretreated with OA at different concentrations (0.5 to 25 µM) for 1 h. LPS was added to OA-pretreated THP-1 macrophages for 48 h at 37 °C in 5% CO_2_–95% air. DMSO was added to control cells. The XTT assay was performed following the instructions of the manufacturer’s kit (Canvax Biotech, Córdoba, Spain) to determine the cell viability. Absorbance was measured at 450 nm. Cell viability was calculated using the equation: (mean OD treated cells/mean OD control cells) × 100.

### 2.9. Determination of Proinflammatory Cytokines and Kinases in LPS-Stimulated THP-1 Macrophages Pretreated with OA

THP-1 macrophages were pretreated for 1 h with OA (0.5, 1, 5, 10 and 25 µM), followed by LPS (100 ng/mL) for 48 h at 37 °C in 5% CO_2_–95% air. DMSO was added to control cells. All experiments were performed in triplicate. Once the incubation period was finished, culture media were collected and the IL-6, IL-1β and TNF-α levels were measured using ELISA kits (Diaclone, Besançon, France; RayBiotech, Norcross, GA, USA; and Cloud-Clone, Katy, TX, USA, respectively). Furthermore, THP-1 macrophages were washed with PBS, scraped off the plates and lysed by sonication twice for 50 s at 50 W. The protein content of the lysate was measured using the Bradford protein assay [32]. Cell lysates were used for measuring the presence of Akt and AMPK-α with ELISA kits (ThermoFisher, Carlsbad, CA, USA). ELISA assays were performed according to the manufacturer’s instructions and absorbance was read at 450 nm.

### 2.10. TRL Stimulation of THP-1-Derived Macrophages and Determination of Proinflammatory Cytokines and Kinases

Appropriate volumes of TRL to achieve a concentration of 25 μg apoB/total protein were added to THP-1 macrophages. PBS (50 µL per mL of cell culture) was added to macrophages as a vehicle in triplicate. After 48 h of incubation at 37 °C in 5% CO_2_–95% air, culture media were collected and THP-1 macrophages were washed with PBS, scraped off the plates and lysed twice for 50 s at 50 W by sonication. The Bradford protein assay [32] was performed to measure the protein content of the lysates.

The levels of IL-6, IL-1β and TNF-α produced in the media were measured by ELISA kits (Diaclone, Besançon, France; RayBiotech, Norcross, GA, USA; and Cloud-Clone, Katy, TX, USA). In addition, ELISA kits were used to analyze the content of Akt, AMPK-α and NF-κB (ThermoFisher, Carlsbad, CA, USA for Akt and AMPK-α; and Cloud-Clone, Katy, TX, USA for NF-κB) using the cell lysates. Absorbance was read at 450 nm.

### 2.11. Oil Red O Staining

THP-1 macrophages were incubated with TRLs at the same conditions as described in Section 2.10. After incubation, macrophages were washed with PBS and treated with 1 mL of isopropanol 60% for 2 min. Once isopropanol was removed, 1 mL of Oil Red O (0.2% (*m*/*v*) in 40% isopropanol/water (*v*/*v*)) was added and cells were incubated at room temperature for 10 min. Oil Red O was removed, and cells were washed with PBS. Finally, 2 mL of glycerol 30% in water (*v*/*v*) was added for preservation for 3–4 days. An inverted microscope Motic AE21 Series (Barcelona, Spain) coupled to a Moticam 2500 5.0M Pixel Live Resolution camera (Motic, Barcelona, Spain) was used for microphotography.

### 2.12. Intracellular Fatty Acid Composition

For the analysis of fatty acids accumulated intracellularly, THP-1 macrophages were incubated at the same conditions as described in Section 2.10. After incubation, cells were washed with PBS, scraped off the plates and lysed twice for 50 s at 50 W by sonication. A direct method of fatty acid methylation was carried out [33]. In brief, 10 µL of 15:0 fatty acid at 1 mg/mL was added as an internal standard to 50 µL of cell lysates in a glass tube. In total, 1.5 mL of methanolic HCl 3N was added and shaken for 30 s. Tubes were heated at 85 °C for 45 min, and after cooling down to room temperature, 0.5 mL of hexane was added to each glass tube, shaken for 30 s and centrifuged at 1000 rpm for 5 min twice. Finally, the upper phase of each tube was collected, evaporated under a stream of nitrogen and concentrated to 75 µL of hexane. An aliquot of 1 µL was injected in a model 5890 series II GC (Hewlett-Packard Co, Avondale, AZ, USA) equipped with a flame ionization detector and a capillary silica column Supelcowax 10 (Supelco Co., Bellefonte, PA, USA) of 60 m length and 0.25 mm internal diameter. Fatty acids were identified by comparison of their retention times against those of standards and quantified by internal standard using peak area integration.

### 2.13. Statistical Analysis

The data were expressed as mean ± standard error of mean (SEM), except olive oil composition, which was expressed as mean ± standard deviation (SD). Values of Akt, AMPK-α, NF-κB and intracellular fatty acids were normalized by the protein content of the lysates. ANOVA followed by Tukey’s test was used for assessing the mean differences among control and experimental groups in the cell experiments. Mean differences of the baseline characteristics of adolescents were assessed by Student’s *t*-test. Statistical analyses were carried out using IBM SPSS Statistics 24.0 (IBM Corp., Armonk, NY, USA) software. GraphPad Prism 6 (San Diego, CA, USA) was used for figures.

## 3. Results

### 3.1. OA Effects on LPS-Stimulated THP-1 Macrophages

#### 3.1.1. Effects of OA Pretreatment on THP-1 Cell Viability

The XTT assay was performed to determine cell viability when treated with OA. Cell viability was not threatened by the presence of OA at all concentrations assayed with a proportion of active cells that remained always above 87%. It was found that both DMSO and LPS, at the applied concentrations, did not affect the viability of THP-1 cells (data are shown in Figure S1).

#### 3.1.2. IL-1β, IL-6 and TNF-α Release in LPS-Stimulated THP-1 Macrophages Pretreated with OA

**Figure 1:**
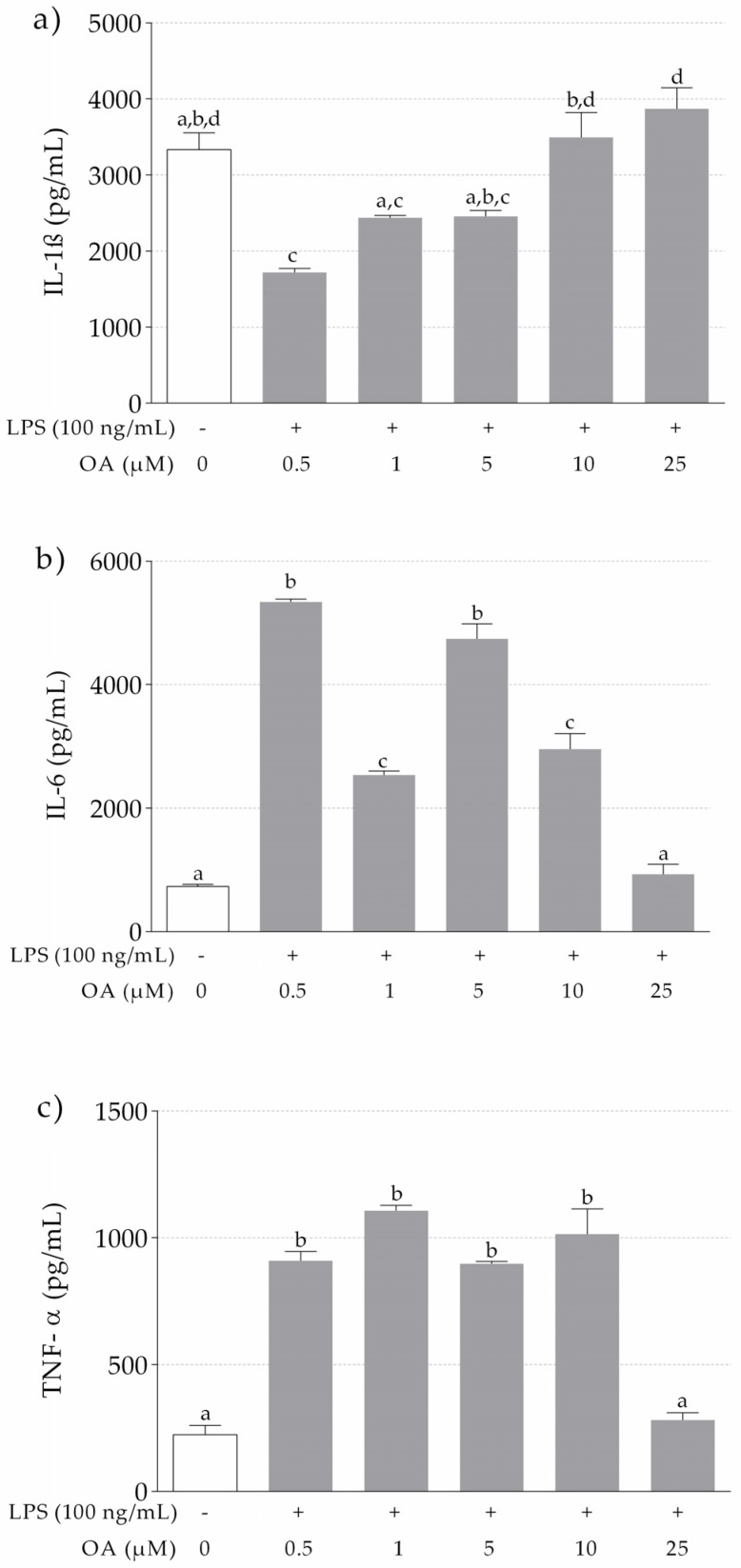
Effects of OA on proinflammatory cytokines production by THP-1 macrophages induced by LPS. Control cells were incubated with DMSO. Experiments performed in triplicate. (**a**) IL-1β; (**b**) IL-6; (**c**) TNF-α. Different letters indicate significant difference (*p* < 0.05) by one-way ANOVA analysis and Tukey’s post hoc test.

Our results show significant higher levels of IL-1β in LPS-stimulated macrophages pretreated with OA at 10 µM and 25 µM, in comparison to lower OA concentrations (Figure 1a). IL-1β levels at the highest OA concentrations were similar to those of non-stimulated cells treated with DMSO only. Concerning IL-6 (Figure 1b) and TNF-α (Figure 1c), a significant increase in the release of these cytokines in THP-1 macrophages treated with LPS was observed in comparison to THP-1 macrophages only exposed to DMSO. However, pretreatment with OA attenuated the release of IL-6 and TNF-α, especially at 25 µM.

#### 3.1.3. Levels of AMPK-α and Akt in LPS-Stimulated and OA-Pretreated THP-1 Macrophages

**Figure 2:**
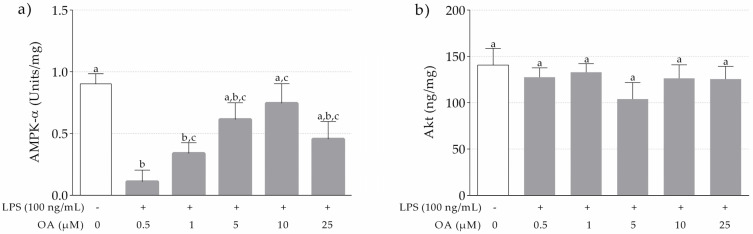
Effects of OA on AMPK-α (**a**) and Akt (**b**) in THP-1 macrophages induced by LPS. Control cells were incubated with DMSO. Experiments performed in triplicate. Different letters indicate significant difference (*p* < 0.05) by one-way ANOVA analysis and Tukey’s post hoc test.

AMPK-α levels in LPS-stimulated THP-1 cells increased dose-dependently up to 10 µM. In contrast, at 25 µM, the concentration was lower than at 10 µM (Figure 2a). The LPS-stimulated THP-1 macrophages pretreated with 0.5 and 1 µM of OA had significantly lower levels of AMPK-α in comparison to non-LPS-stimulated THP-1 macrophages treated with DMSO. Therefore, 10 µM was the concentration at which OA was more effective in attenuating the dephosphorylation of AMPK-α induced by LPS. No significant differences were observed in the levels of Akt (Figure 2b).

### 3.2. Postprandial Clinical Trial. Activation of THP-1 Macrophages with Human TRL

#### 3.2.1. Anthropometric and Biochemical Data of Adolescents at Baseline

Table S2 shows the anthropometric and biochemical characteristics of adolescents, whose data were assessed prior to the postprandial trial. All data were within normal limits.

#### 3.2.2. Effects of Human TRLs on the Production of Proinflammatory Cytokines in THP-1 Macrophages

**Figure 3:**
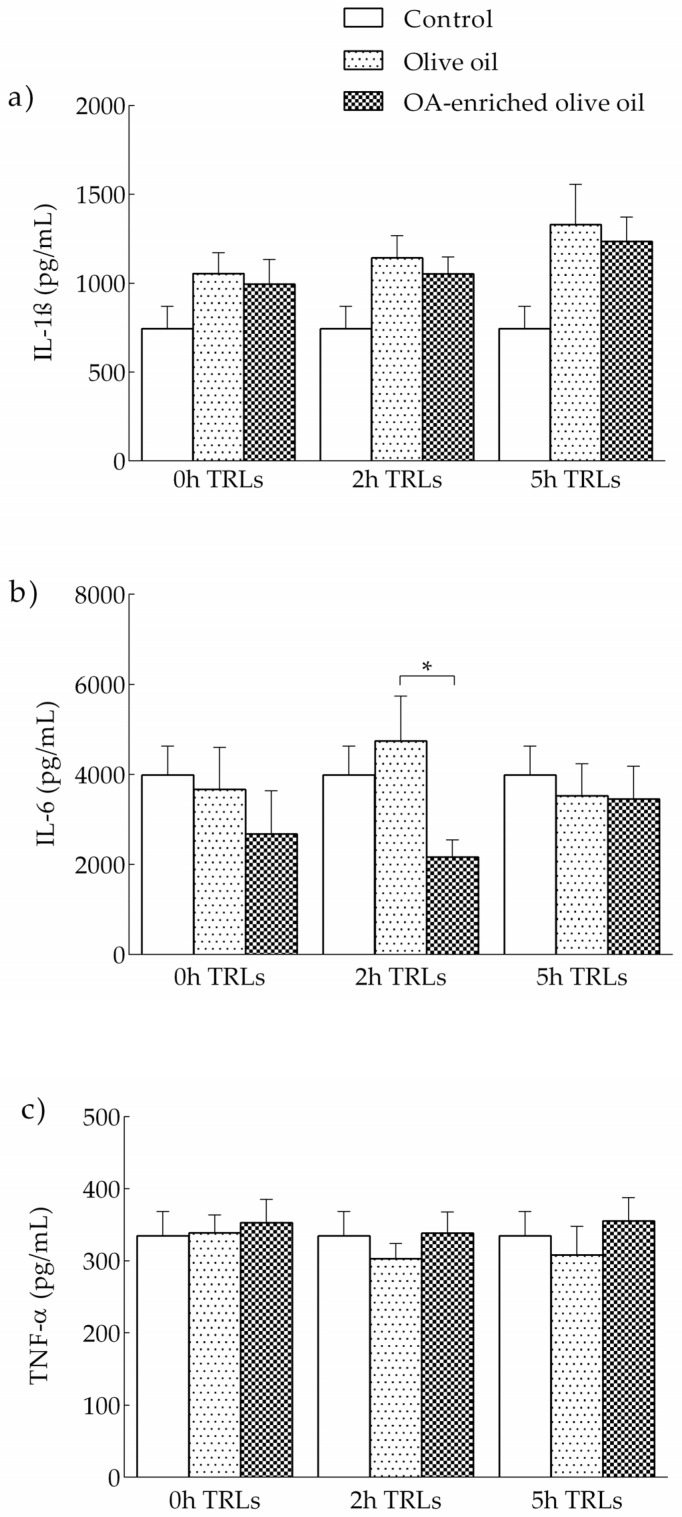
TRL production of proinflammatory cytokines by THP-1 macrophages incubated with TRLs obtained from healthy adolescents before, and at 2 and 5 h after the intake an OA-enriched olive oil or non-functional olive oil. Control cells incubated with PBS. (**a**) IL-1β; (**b**) IL-6; (**c**) TNF-α. * *p* < 0.05.

TRL-induced production of the proinflammatory cytokines IL-1β, IL-6 and TNF-α on THP-1 macrophages is shown in Figure 3. At the same time point, cells stimulated with 2 h postprandial TRL in adolescents that ingested the OA-enriched olive oil produced lower levels of IL-6 than those treated with 2 h postprandial TRL after the intake of the non-functional olive oil. Non-statistically significant differences were observed for IL-1β and TNF-α.

We did not find statistically significant differences for any variable among the different time points in the macrophages incubated with either olive oil derived TRLs or OA-enriched olive oil TRLs.

#### 3.2.3. Effects of Human TRLs on the Levels of Kinases and NF-κB in THP-1 Macrophages

**Figure 4:**
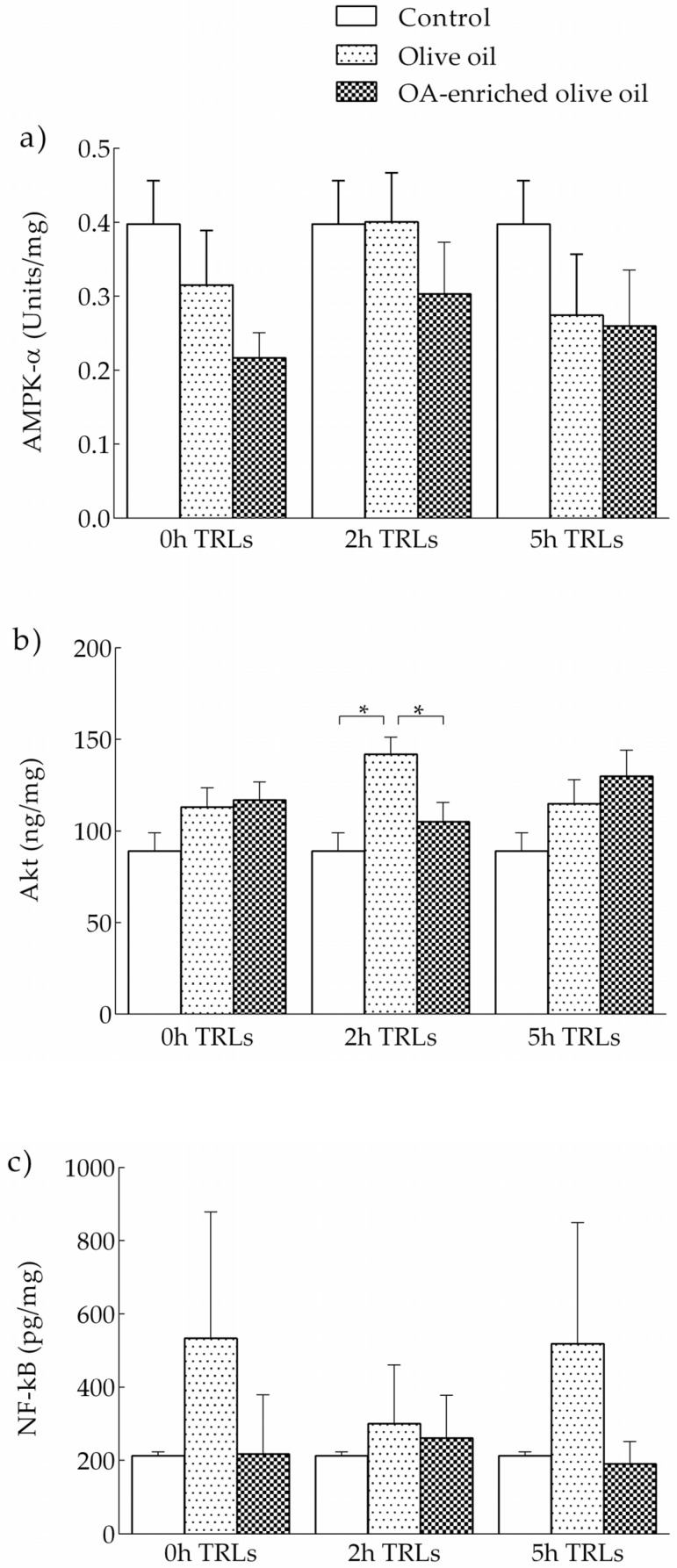
Levels of AMPK-α (**a**), Akt (**b**) and NF-κB (**c**) in THP-1 macrophages incubated with TRL obtained from healthy adolescents before, and at 2 and 5 h after the intake an OA-enriched olive oil or non-functional olive oil. Control cells incubated with PBS. * *p* < 0.05.

The levels of AMPK-α and Akt proteins after incubation of THP-1 macrophages with postprandial TRLs are shown in Figure 4. At the same time point, levels of Akt were higher in macrophages stimulated with 2 h postprandial TRL of adolescents that ingested the non-functional olive oil compared to PBS. In contrast, THP-1 macrophages stimulated with 2 h postprandial TRL after the intake of the functional olive oil presented levels of Akt similar to PBS. No significant differences in levels of AMKP-α and NF-κB were observed.

No significant differences for kinases and NF-κB were observed at the different time points in either the macrophages incubated with olive oil-derived TRLs or with OA-enriched olive oil-derived TRLs.

#### 3.2.4. Intracellular Lipid Accumulation

**Figure 5:**
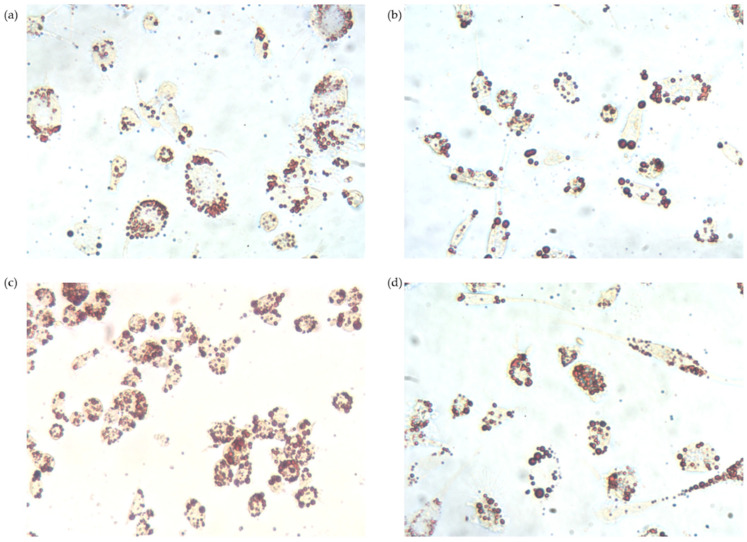
Oil red O microphotographs of THP-1 macrophages incubated for 48 h in the presence or absence (control) of TRLs obtained before, and at 2 and 5 h after the consumption of the olive oils. (**a**) Control; (**b**) 0 h TRL; (**c**) 2 h TRL; (**d**) 5 h TRL.

Figure 5 shows microphotographs of intracellular lipids stained with Oil Red O. The lipid content was higher in TRL-treated cells, independent of the presence of OA. The fatty acid content of total lipids accumulated intracellularly by THP-1 macrophages after being incubated for 48 h in the absence (control) or presence of TRLs isolated at baseline, 2 and 5 h after the consumption of the OA-enriched or non-functional olive oils are shown in Table 1. A higher oleic acid (18:1) content was found in cells incubated with TRLs obtained 2 h after the intake of the experimental meals compared to control but not compared to the other time points studied. For the rest of the fatty acids, no differences were found compared to either the control or other time points.

## 4. Discussion

In the present work, we evaluated the ability of OA to attenuate the overproduction of proinflammatory cytokines and to modulate signaling pathways in THP-1 macrophages stimulated by LPS, as well as its activity when forming part of human TRLs obtained after the intake of an olive oil enriched in the triterpene. The main findings presented here are (i) pretreatment with OA attenuated the LPS-induced overproduction of pro-inflammatory cytokines, especially of IL-6, but also TNF-α, in THP-1 macrophages; (ii) levels of AMPK-α were increased by OA in a dose-dependent manner in LPS-stimulated THP-1 macrophages; and (iii) OA-enriched olive oil alleviated the levels of IL-6 and Akt in THP-1 macrophages stimulated with 2 h postprandial TRLs.

LPS is widely used in experiments to induce an inflammatory response, and numerous studies have shown an overproduction of IL-1β, IL-6 and TNF-6 in THP-1 cells stimulated by LPS [34,35,36]. In our study, the activation of THP-1 macrophages with LPS produced an abundant release of IL-6, IL-1β and TNF-α in the cell culture supernatants. However, pretreatment with OA attenuated IL-6 levels, especially at 25 µM. At that concentration, OA was also able to reduce TNF-α release by the cells. Similar results were reported by Castellano et al. [37] in LPS-activated BV-2 microglia, where the overproduction of IL-6 was attenuated in a dose-dependent manner by OA. In contrast, unexpected results were reported in our study for LPS-induced production of IL-1β, since a dose-dependent increase of this cytokine by OA was observed. Interestingly, LPS-stimulated THP-1 macrophages pretreated with the highest concentrations of OA released similar values of IL-1β than the control. It has been reported that DMSO induces IL-1β secretion in THP-1 macrophages by activating the enzyme caspase-1 [38,39]. This could partly explain our findings, since OA was dissolved in DMSO, and the cells that received the higher doses of OA also received higher DMSO volumes. Nevertheless, DMSO is a common solvent for water-insoluble substances, such as OA, in this kind of experiment [39,40].

The effects of OA on proinflammatory cytokines produced by THP-1 macrophages, especially IL-6, might be explained by the modulatory role of AMPK on inflammation processes. In order to become active, AMPK needs to be phosphorylated at its α-subunit (Thr172 residue) [41,42]. It has been reported that LPS induces the dephosphorylation of AMPK [42]. Therefore, to achieve a better understanding of the anti-inflammatory ability of OA in THP-1 macrophages, the levels of phosphorylated AMPK-α were measured. A significant reduction of LPS-induced dephosphorylation of AMPK-α was observed at 10 µM of OA. In line with our study, Liu et al. [43] reported that δ-oleanolic acid could stimulate the phosphorylation of AMPK in THP-1 macrophages. In the same way, Matumba et al. [44] reported an increase in the AMPK gene expression and a reduction of IL-6 and TNF-α gene expression in high-fructose diet-fed rats treated with OA. Furthermore, the application of an OA derivative, 3-acetyl-oleanolic acid, to rats fed with a high-fructose diet has been shown to significantly increase the phosphorylation of AMPK in the liver tissues [45]. Therefore, OA seems to have a key role in the alleviation of inflammatory status through regulating AMPK phosphorylation.

Indeed, AMPK is believed to be one of the main mechanisms of the biological activity of OA, as well as other triterpenic compounds. OA modulates glucose uptake, fatty acid oxidation in the muscle and fatty acid synthesis and gluconeogenesis in the liver by stimulating AMPK phosphorylation [46]. Another reported signaling pathway underlying the metabolic effects of OA is PI3K/Akt. For instance, OA and its derivatives have been shown to stimulate Akt in vascular smooth muscle cells [47], and to relieve inflammation in different experimental models [48,49,50]. However, not all OA derivatives have the same inflammatory effect through Akt. Jin et al. [51] prepared eleven oxooleanolic acid derivatives using OA as starting compounds for structural modification, and found that only two of them exerted significant anti-inflammatory effects on BV-2 cells by the activation of NF-κB, MAPKs and PI3K/Akt. In addition, in bone marrow macrophages, OA showed no significant inhibitory effects on receptor activator of nuclear factor kappa-B ligand-activated expression of NF-κB, p38, JNK and Akt [52]. However, in our study, OA was unable to significantly reduce Akt levels. There are three homologous Akt isoforms that are activated by similar mechanisms, Akt1, Akt2 and Akt3 [53]. In THP-1 macrophages, Shiratsuchi and Basson [54] found that Akt2 but not Akt1 and Akt3 mediated phagocytosis stimulated by pressure. Since we measured total Akt, the absence of a clear effect might be related to opposing roles of the different isoforms. In the same way, a Mg-based alloy inhibited the inflammatory response of THP-1 cell-derived macrophages through the inhibition of Akt1 but not total Akt [55]. Therefore, further studies are needed to clarify the participation of Akt isoforms in the regulation of proinflammatory cytokines in THP-1 macrophages by OA.

Postprandial TRL participates in the atherosclerotic process through promoting foam cell formation from macrophages, in which the secretion of pro-inflammatory substances plays an important role [56,57]. The induction of THP-1 macrophages in our study with 2 h postprandial TRLs derived from the OA-enriched olive oil showed significant lower levels of IL-6 but not IL-1β and TNF-α, compared to the TRLs derived from olive oil. Our results have some similarities with the study performed by Graham et al. [58] with postprandial TRLs from healthy adults. They reported lower levels of IL-6 in THP-1 cells incubated with 4 h TRLs derived from pomace olive oil, which was attributed to the presence of OA in the oil. In addition, and in agreement with our results, chylomicron remnants, a form of TRL, but not native chylomicrons or oxLDL, did not increase IL-1β and TNF-α in vascular smooth muscle cells, which are also involved in atherogenesis, despite increased monocyte chemoattractant protein (MCP)-1 mRNA expression [59,60].

The potential role of OA might be due to its repressive action on NF-κB, which has been shown to be activated by TRLs [61]. Although we did not find significant influences of TRL on NF-κB, levels of this factor were slightly lower in macrophages treated with postprandial TRL derived from OA-enriched olive oil. The effect of TRLs on NF-κB is highly dependent upon their fatty acid composition. De Pascale et al. [62] used lab-made TRLs to induce foam cell formation in PMA-activated THP-1 macrophages, and found a lowering effect on the NF-κB binding of polyunsaturated fatty acids, but not by monounsaturated fatty acids. Since the TRLs employed in the present study were obtained after the intake of olive oil, which is rich in monounsaturated fatty acids, the lack of a statistically significant effect of these particles on NF-κB might be related to their fatty acid composition, with no intervention of OA. In contrast, at 2 h, TRLs obtained from the control olive oil significantly increased Akt levels, which were ameliorated when these lipoproteins were isolated after the intake of the OA-enriched oil. The application of postprandial very low-density lipoproteins (VLDL) to peripheral blood mononuclear cells and THP-1 monocytes by den Hartigh et al. [63] produced an overexpression of Akt phosphorylation, which, in contrast to our study, was accompanied by increased gene expression levels of TNF-α and IL-1β. Unfortunately, these authors did not measure IL-6 in the culture medium.

The intracellular lipid accumulation after administering postprandial TRLs into THP-1 macrophages varies depending on several factors, including the postprandial time at which TRLs are isolated [56]. Cabello-Moruno et al. [56], Graham et al. [58], Perona et al. [64] and Cabello-Moruno et al. [65] have repeatedly showed that the intracellular lipid composition is very dependent on the composition of TRLs, which in turn, reflects that of the ingested oils. Nevertheless, we previously demonstrated that minor components of pomace olive oil can also modify postprandial TRL composition and the clearance of TG molecular species of postprandial TRL [66]. Therefore, it was reasonable to suggest that the presence of OA in TRL would affect the intracellular lipid composition of THP-1 macrophages. However, we only found slight changes in the oleic acid content of intracellular lipids. This is, in fact, in agreement with previous reports. In the study by Graham et al. [58], no differences were found in the intracellular lipid content when THP-1 macrophages were incubated with TRLs obtained 2 h or 4 h after the intake of virgin, pomace and OA-enriched pomace olive oil.

The present study shows that OA is capable of alleviating the proinflammatory cytokines overproduction in THP-1 macrophages, probably due to the enhancement of AMPK-α levels. The effect was also observed when cells were treated with OA as part of postprandial TRLs, as obtained from the blood of adolescents after the intake of a functional olive oil enriched with the triterpene. Therefore, OA has potential anti-atherogenic activity and might be considered to be used in functional foods, at least in adolescents.

This work has some strengths and limitations. For the first time a formulation of olive oil enriched with the triterpene OA has been used in a postprandial trial performed on a population of adolescents. TRLs isolated from adolescent blood and different concentrations of OA were supplied to THP-1 macrophages, obtaining interesting results that must be interpreted with caution, due to its difficult generalization to other cells or whole organisms. Nevertheless, our results offer new data on OA effects on signaling pathways, allowing a better understanding of the mechanisms of OA action.

## 5. Conclusions

In summary, pretreatment of THP-1 macrophages with OA attenuated the overproduction of proinflammatory cytokines, especially of IL-6, and enhanced the AMPK-α levels in a dose-dependent manner. Therefore, we conclude that OA has potential to treat insulin resistance due to its capability to alleviate the inflammatory response through modulating the activity of AMPK-α. In regard to the results reported on the postprandial trial, TRLs obtained from adolescents 2 h after the ingestion of the functional olive oil led to lower levels of IL-6 and Akt in macrophages. These results suggest that OA could have the potential to be used as a lipid-based formulation in functional olive oils to prevent and treat insulin resistance, atherogenic impairments and the different metabolic factors underlying MetS in adolescents. However, further cell experiments using OA and clinical trials with OA-enriched olive oil must be performed in order to obtain more solid data to allow the future use of OA as a nutraceutical in the preventive strategies of MetS and insulin resistance.

## Figures and Tables

**Table 1:** Intracellular fatty acid composition (μg/mg cell protein) of total lipids in THP-1 macrophages after incubation with TRLs derived from two different olive oils.

		0 Hour		2 Hours		5 Hours	
Fatty Acids	Control	Olive Oil	OA-Enriched Olive Oil	Olive Oil	OA-Enriched Olive Oil	Olive Oil	OA-Enriched Olive Oil
12:0	41.46 ± 3.41	41.68 ± 4.26	38.93 ± 3.23	42.10 ± 5.51	41.02 ± 2.21	35.63 ± 4.93	40.06 ± 6.80
14:0	45.00 ± 3.33	48.13 ± 4.26	42.41 ± 2.38	48.18 ± 4.30	40.71 ± 3.10	40.94 ± 2.95	41.41 ± 4.56
16:0	95.68 ± 16.74	158.11 ± 26.64	151.96 ± 26.97	171.74 ± 19.10	148.57 ± 24.76	130.98 ± 20.06	180.53 ± 48.50
18:1	60.16 ± 6.51	119.58 ± 24.85	104.55 ± 22.00	126.60 ± 13.02 *	119.88 ± 19.83	102.02 ± 14.42	145.63 ± 44.69
18:2	46.68 ± 6.07	45.22 ± 4.12	40.83 ± 3.43	56.26 ± 11.56	41.65 ± 3.45	37.49 ± 2.99	40.59 ± 4.35
20:4	41.74 ± 1.30	51.22 ± 16.64	63.37 ± 17.74	46.04 ± 4.73	43.44 ± 6.23	42.60 ± 11.64	35.02 ± 1.65
Others	79.37 ± 9.00	ND	59.97 ± 26.24	ND	ND	ND	155.12 ± 0.57

Data are presented as mean ± SEM. Controls performed in triplicate. Experimental groups (*n* = 11). * *p* < 0.05 vs. control. Statistical differences were analyzed among control, olive oil and the OA-enriched olive oil at the same time point and at different time points. ND, not detected.

## Data Availability

The data presented in this study are available on request from the corresponding author.

## Supplementary Materials

The following are available online at https://www.mdpi.com/article/10.3390/nu13103471/s1, Table S1: Composition of the olive oil used in the study, Figure S1: Cell viability in OA-pretreated THP-1 macrophages at different concentrations as measured by XTT, Table S2: Baseline anthropometric and biochemical characteristics of adolescents according to the olive oil ingested at breakfast.
