# Supplementary material for: Oleanolic Acid-Enriched Olive Oil Alleviates the Interleukin-6 Overproduction Induced by Postprandial Triglyceride-Rich Lipoproteins in THP-1 Macrophages
Source: Nutrients. 2021 Sep 29;13(10):3471. doi: 10.3390/nu13103471 (PMC8537268; doi:10.3390/nu13103471)
Supplement: Supplementary file 1 [file nutrients-13-03471-s001.zip › Tables S1 and S2.pdf]

Table S1. Composition of the olive oil used in the study

Component	Mean ± SD
Fatty acids (%)	
Palmitic (16:0)	11.0 ± 0.4
Palmitoleic (16:1 n-7)	1.0 ± 0.1
Estearic (18:0)	3.2 ± 0.1
Oleic (18:1 n-9)	75.7 ± 0.2
Linoleic (18:2, n-6)	7.0 ± 0.0
α-linolenic (18:3 n-3)	0.3 ± 0.1
Arachidic (20:0)	0.4 ± 0.0
Gadoleic (20:1 n-11)	0.6 ± 0.1
Total phenolics (μg/g oil)	60.7 ± 0.8
Hydroxytyrosol and derivatives	21.9 ± 0.2
Tyrosol and derivatives	38.6 ± 0.3
Lignanes	0.4 ± 0.0
Flavonoids	0.3 ± 0.1
Simple phenols	0.1 ± 0.0
Total sterols (μg/g oil)	1295.3 ± 3.9
β-sitosterol	1124.0 ± 1.9
δ5-avenasterol	46.5 ± 0.7
Campesterol	44.0 ± 0.1
Stigmasterol	11.7 ± 0.1
Clerosterol	14.2 ± 0.0
δ5,24-stigmastadienol	14.9 ± 0.7
δ5,23-stigmastadienol	9.1 ± 0.0
Total tocopherols (μg/g oil)	263.8 ± 4.1
α-tocopherol	230.6 ± 0.8
Triterpenoids (μg/g oil)	
Erythrodiol	37.6 ± 0.1
Uvaol	3.6 ± 0.8
Oleanolic acid (control/enriched oils)	3.8 ± 0.1 / 610.4 ± 16.2
Maslinic acid	4.8 ± 0.1
Total chlorophylls (μg/g oil)	1.1 ± 0.1
Total carotenoids (μg/g oil)	1.5 ± 0.1

SD, Standard deviation

Table S2. Baseline anthropometric and biochemical characteristics of adolescents according to the olive oil ingested at breakfast.

Variables	Olive oil n = 11	OA-enriched olive oil n = 11
Gender (%)		
Boys	2 (18.2)	3 (27.3)
Girls	9 (81.8)	8 (72.7)
Mean age (years)	16.00 ± 0.00	16.18 ± 0.12
Body weight (kg)	57.26 ± 2.74	59.57 ± 2.42
BMI (kg/m^2)	20.67 ± 0.87	21.10 ± 0.67
Body fat (%)	21.43 ± 2.36	21.33 ± 2.18
WC (cm)	68.55 ± 1.90	70.64 ± 2.08
SBP (mmHg)	124.36 ± 3.16	122.91 ± 3.20
DBP (mmHg)	76.73 ± 2.91	76.91 ± 2.05
TG (mg/dL)	56.45 ± 4.81	58.82 ± 4.08
TC (mg/dL)	172.10 ± 4.57	168.09 ± 5.81
LDL-c (mg/dL)	115.36 ± 6.29	96.64 ± 8.59
HDL-c (mg/dL)	45.18 ± 3.78	59.10 ± 6.88
Glucose (mg/dL)	70.73 ± 1.59	72.64 ± 1.88
Insulin (μU/ml)	7.27 ± 0.93	7.50 ± 1.65
HOMA-IR	1.27 ± 0.17	1.32 ± 0.29
Pubertal stage (%)[1]		
Tanner stage 1/2	0 (0)	0 (0)
Tanner stage 3	3 (27.3)	1 (9.1)
Tanner stage 4	6 (54.5)	4 (36.4)
Tanner stage 5	2 (18.2)	6 (54.5)

Data are presented as mean ± SEM or %. Significant differences were not found. BMI, body mass index; WC, waist circumference; SBP, systolic blood pressure; DBP, diastolic blood pressure; TG, triglycerides; TC, total cholesterol; LDL, low-density lipoprotein cholesterol; HDL-c, high-density lipoprotein cholesterol; HOMA-IR, homeostatic model assessment of insulin resistance. [1]Based on self-reported Tanner stages.
