# Supplementary figures and images for: Oleanolic Acid-Enriched Olive Oil Alleviates the Interleukin-6 Overproduction Induced by Postprandial Triglyceride-Rich Lipoproteins in THP-1 Macrophages
Source: Nutrients. 2021 Sep 29;13(10):3471. doi: 10.3390/nu13103471 (PMC8537268; doi:10.3390/nu13103471)

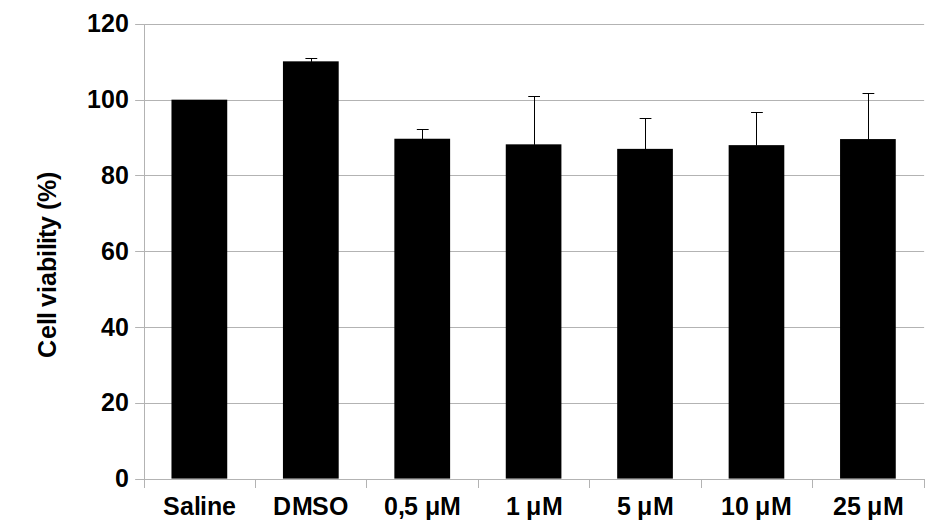

Supplement: Supplementary file 1 [file nutrients-13-03471-s001.zip › Figure S1.png]
